# Preclinical evaluation of Mito-LND, a targeting mitochondrial metabolism inhibitor, for glioblastoma treatment

**DOI:** 10.1186/s12967-023-04332-y

**Published:** 2023-08-07

**Authors:** Tongxuan Guo, Changyong Wu, Lingni Zhou, Junhao Zhang, Wanzhou Wang, Yang Shen, Ludong Zhang, Mingshan Niu, Xu Zhang, Rutong Yu, Xuejiao Liu

**Affiliations:** 1 Insititute of Nervous System Diseases, Xuzhou Medical University, Xuzhou, Jiangsu China; 2 Department of Neurosurgery, Affiliated Hospital of Xuzhou Medical University, Xuzhou, Jiangsu China; 3 Blood Diseases Institute, Xuzhou Medical University, Xuzhou, Jiangsu China

**Keywords:** GBM, Mito-LND, Mitochondrial metabolism, Raf/MEK/ERK signaling pathway, Tumor cell proliferation

## Abstract

**Background:**

Glioblastoma (GBM) is a brain tumor with the highest level of malignancy and the worst prognosis in the central nervous system. Mitochondrial metabolism plays a vital role in the occurrence and development of cancer, which provides critical substances to support tumor anabolism. Mito-LND is a novel small-molecule inhibitor that can selectively inhibit the energy metabolism of tumor cells. However, the therapeutic effect of Mito-LND on GBM remains unclear.

**Methods:**

The present study evaluated the inhibitory effect of Mito-LND on the growth of GBM cells and elucidated its potential mechanism.

**Results:**

The results showed that Mito-LND could inhibit the survival, proliferation and colony formation of GBM cells. Moreover, Mito-LND induced cell cycle arrest and apoptosis. Mechanistically, Mito-LND inhibited the activity of mitochondrial respiratory chain complex I and reduced mitochondrial membrane potential, thus promoting ROS generation. Importantly, Mito-LND could inhibit the malignant proliferation of GBM by blocking the Raf/MEK/ERK signaling pathway. In vivo experiments showed that Mito-LND inhibited the growth of GBM xenografts in mice and significantly prolonged the survival time of tumor-bearing mice.

**Conclusion:**

Taken together, the current findings support that targeting mitochondrial metabolism may be as a potential and promising strategy for GBM therapy, which will lay the theoretical foundation for further clinical trials on Mito-LND in the future.

**Supplementary Information:**

The online version contains supplementary material available at 10.1186/s12967-023-04332-y.

## Background

Glioblastoma (GBM) is the most common primary intracranial malignant brain tumor [1]. Currently, the standard therapy of GBM is still operation for maximum resection of the tumor, accompanied by postoperative adjuvant radiotherapy and chemotherapy [2]. However, due to invasion, the postoperative recurrence rate is high, and the prognosis of patients is poor [3]. Temozolomide (TMZ) has been applied in clinical practice for > 20 years and is currently the first-line clinical chemotherapy drug [4]. However, most GBM patients are insensitive to TMZ, which does not significantly improve patients’ survival [5]. Thus, identifying new therapeutic targets and developing small-molecule targeted drugs to provide effective therapies and strategies for the treatment of GBM is an urgent requirement.

Although tumor cells rely primarily on aerobic glycolysis to produce adenosine triphosphate (ATP) (Warburg effect) [6], many studies have shown that mitochondria play a crucial role in tumorigenesis [7]. In addition to supplying energy to cells, mitochondria are involved in major life activities, such as tumor anabolism, cell senescence, apoptosis, and signal transduction [8]. The regulation of glycolysis of tumor cells by inhibiting the respiratory chain of mitochondria is a critical approach against tumors [9]. IACS-010759, a small molecule inhibitor, attenuates mitochondrial oxidative phosphorylation by inhibiting mitochondrial complex I activity, thereby inhibiting GBM and the growth of leukemia cells [10].

Moreover, mitochondrial metabolism produces some metabolic by-products, such as reactive oxygen species (ROS), that promote apoptosis [11]. Excessive ROS can promote DNA damage and induce mitochondrial apoptosis [12]. However, cancer cells with high ROS levels close to the toxicity threshold are more susceptible to pro-oxidative agent-mediated apoptosis than normal cells [13]. Some studies suggest that drugs inhibiting the mitochondrial complex I, such as metformin, play a key role in anti-tumor activity [14]. Reduced mitochondrial complex I activity leads to electron leakage, which in turn leads to excessive ROS accumulation and mitochondrial dysfunction [15]. Thus, mitochondrial metabolism is becoming a promising target for the development of novel anti-tumor drugs.

Lonidamine (LND) is a small molecule inhibitor of energy metabolism that selectively inhibits tumor cells [16]. LND can impair glycolytic pathway and/or interfere with the pyruvate carrier and plasma membrane monocarboxylate transporters of the MCT family [17]. Besides, it can also promote cell death which was potentiated by its suppression of cancer cell energy metabolism [18]. LND has undergone clinical trials in combination with standard-of-care chemotherapeutics for multiple cancers [19 20]. Especially, combination treatment of LND and diazepam has got into Phase II clinical trial of recurrent glioblastoma multiforme [18, 21]. LND and diazepam, acting on two distinct mitochondrial sites involved in cellular energy metabolism, may exert a cytostatic effect on tumor growth. The combination is well tolerated, but to have limited efficacy [18]. Due to the negative potential of the mitochondrial outer membrane [22], the covalently linked lipophilic cation triphenylphosphine (TPP+) selectively interacts with the extracorporeal membrane and is retained on the surface of the mitochondrial membrane [23]. Therefore, Mito-LND is synthesized by connecting LND with TPP+, which can target the mitochondria. More importantly, the IC_50_ values for inhibiting cell proliferation were 188- and 300-fold lower for Mito-LND than LND [24]. In addition, Mito-LND is one of the least toxic mitochondrial targeting cations. Previous study has shown that Mito-LND inhibits AKT/mTOR pathway and promotes autophagy to inhibit lung cancer [24]. However, whether Mito-LND has an anti-tumor effect on glioblastoma is yet to be elucidated.

In the present study, we determine the effects of Mito-LND on the growth of GBM cells and GBM xenografts. Furthermore, we elucidate the putative mechanism that Mito-LND inhibits GBM proliferation and induces apoptosis.

## Methods

### Culture of cell lines

The human cell lines (LN229, U251, T98G, and U87) and normal human astrocyte (HA1800) used in this study were cultured and maintained in Dulbecco’s Modified Eagle Medium (DMEM) supplemented with 10% fetal bovine serum (FBS) and grown in a 37 ℃ moist incubator containing 5% CO_2_. Normal human astrocyte (NHA) was grown in astrocyte medium (ScienCell; Cat No.1801) supplemented with penicillin/streptomycin, 2% FBS and astrocyte growth supplement. The GSC cell line was cultured in Neurobasal^™^ Medium containing basic fibroblast growth factor, EGF, B27 supplement, haprin, L-glutamine, and N2 supplement to form a GSC-rich neurosphere culture.

### Reagents and antibodies

Mito-LND, N-Acetylcysteine (NAC) and ERK1/2 activator C16-PAF were purchased from MedChemExpress (Shanghai, China). Mito-LND and C16-PAF were dissolved in DMSO to generate stock solution (10 mM), and N-Acetylcysteine was dissolved in DMSO to produce stock solution (500 mM). These inhibitors were diluted to different concentrations in DMEM medium before use.

Primary antibodies against c-p-Raf (#9421), p-MEK1/2 (#9154), p-p90RSK (#9346), ERK1/2 (#4695), p-ERK1/2 (#4376), CDC2 (#28,439), CDK4 (#12,790), CDK6(#3136), Bax (#2772), Bcl-2 (#4223), cyclin D1 (#2922), cyclin B1 (#4138), GAPDH (#97,166) were purchased from Cell Signaling Technology (CST, MA, USA).

### CCK-8 assay

Cell counting kit-8 (CCK-8, Vicmed, Jiangsu, China) was used for evaluating cell viability. GBM or GSC cells were seeded on 96-well plates with 4000 cells per well, and each group was repeated with 3 duplicate wells. After overnight culture, different concentrations of Mito-LND (0–5 µΜ) were added and treated for 72 h. Then, 10 µL CCK-8 solution were added to each well. After incubation for 30 min without light, absorbance was measured at 450 nm wavelength using a microplate reader.

### In vitro tumorsphere formation assay

GSC1 and GSC2 cells were seeded in 96-well plate at 1000 cells per well. The cells were cultured in NeurobasalTM medium containing a certain concentration of Mito-LND or DMSO, and the formation of tumor spheres was assessed under a microscope after 10 days. Tumorspheres with more than 50 cells were scored, and the number of tumorspheres in each well was counted.

### EdU incorporation assay

EdU Cell Proliferation Detection Kit (Abbkine, Hubei, China) was used to evaluate cell proliferation. Each well was seeded with 10,000 cells in 96-well plates and incubated overnight. Different concentrations of Mito-LND (0–2.5 µΜ) were added and incubated for 12 h. Then, cells were incubated with 10 µM EdU for an additional 2 h and fixed with 4% paraformaldehyde for 30 min. After washing with phosphate-buffered saline (PBS), the cells were treated with 0.5% Triton X-100 for 10 min. Finally, 100 µL Click-iT was added to each well, and the reaction was incubated for 30 min, followed by DAPI staining for 15 min. After three washes with PBS, the images were captured under the inverted fluorescence microscope (Olympus, Tokyo, Japan).

### Colony formation assay

LN229 or U251 cells were seeded in 6-well cell culture plates at 800 cells/well. The cells were incubated overnight, allowing adherence of cells to the plate, followed by the addition of different Mito-LND concentrations (0–2.5 µΜ), and 0.1% DMSO was added to the control wells. After treatment for 12 h, the cells were cultured in the medium without drugs for 14 days. The cells were washed with PBS and fixed with methanol for 30 min, and then were stained with 0.1% crystal violet for 30 min. Cell colonies were observed, photographed, and counted.

### Cell cycle and cell apoptosis assays

LN229 or U251 cells were seeded in 6-cm culture dishes, and different concentrations of Mito-LND (0–2.5 µΜ) were added and incubated for 24 h. Subsequently, the cells were collected for cell cycle analysis and fixed with 70% ice-cold ethanol overnight. After two PBS washes, cells were stained with propidium iodide (PI)/RNase solution for 15 min. For cell apoptosis, cells were washed twice with cold PBS and stained using Annexin V-FITC/PI apoptosis detection kit (Kaiji, Jiangsu, China). The cell cycle distribution and apoptosis were analyzed by flow cytometry and analyzed by flow cytometry software (BD Biosciences).

### Caspase-glo 3/7 activity assay

LN229 and U251 cells were seeded into 96-well plates and treated with different concentrations of Mito-LND (0–2.5 µΜ) for 24 h. Caspase-Glo3/7 enzymatic activities were measured according to the manufacturer’s protocol (Promega, Madison, USA). Briefly, 100 µL of Caspase-Glo 3/7 reagent was added to the wells. After 30 min, 200 µL reaction was transferred to white-walled luminometer plates. The luminescence in each well was detected by the GloMax Luminometer.

### ROS assay

LN229 and U251 cells were seeded in 6-cm culture dishes with 100,000 cells/well. After treatment with 0.1% DMSO and Mito-LND (2.5 µΜ) for 24 h, DCFH-DA probe (Beyotime, Jiangsu, China) was added and incubated 37 °C for 30 min. The probe that did not enter the cells was rinsed two times with PBS, and Rosup group was used as the positive control. The cells were digested with trypsin, and the reaction was terminated with a serum-free medium. The cells were transferred to flow tubes. The proportion of ROS-positive cells was detected by flow cytometry, and the data were analyzed by FlowJo-V10 software (TreeStar Inc.).

### Mitochondrial complex I activity

To assess mitochondrial complex I activity, a CheKine™ Micro Mitochondrial complex I Activity Assay Kit (Abbkine, Hubei, China) was used to extract cell mitochondria according to the manufacturer’s protocol. LN229 and U251 cells were treated with Mito-LND at different concentrations (0–2.5 µΜ) for 6 h. The microplate reader was preheated, and the wavelength was adjusted to 340 nm. A volume of 10 µL sample (mitochondrial complex I), 15 µL working reagent VI, and 200 µL working reagent were added to 96-well UV microplate successively. After mixing, the initial absorption value A1 at 0 min and the absorption value A2 after 2 min were immediately recorded at 340 nm, and DA = A1 − A2. The formula was used to calculate the mitochondrial complex I activity (U/10^4^ cells) = 1.46×DA (U: Consumption of 1 nmol NADH per 10,000 cells/min is defined as U), and the values were recorded and analyzed.

### Mitochondrial membrane potential assay (JC-1)

LN229 and U251 cells were treated with different concentrations of Mito-LND (0-2.5 µΜ) for 24 h. The changes in mitochondrial membrane potential were determined by JC-1-Mitochondrial Membrane Potential Assay Kit (Abcam, Shanghai, China). The medium was discarded, and the cells were washed three times with PBS. The JC-1 dye was thawed at 4 °C and diluted with medium from light to the final concentration of 2 µM. Then, 100 µL of diluted JC-1 dye was added to each well, and the reaction was incubated at 37 °C for 15 min. After washing two times with 1× buffer, fresh medium was added. The images were acquired under the fluorescence microscope (Olympus, Tokyo, Japan).

Quantitative analysis was performed on a microplate reader. The fluorescence intensity of aggregates (red light) was determined with excitation wavelength of 528 nm and emission wavelength of 560 nm. The fluorescence intensity of the monomer (green light) was determined with excitation wavelength 485 nm and emission wavelength 530 nm. The fluorescence values were analyzed statistically. Carbonyl cyanide-4-(trifluoromethoxy)phenylhydrazone (FCCP) (mitochondrial uncoupling agent) was used as the positive control.

### Western blotting

LN229 or U251 cells were treated with Mio-LND (0–2.5 µΜ) for 24 h, and then the total protein was collected for immunoblot analysis as previously described [25]. The protein expression levels of p-c-Raf, p-MEK1/2, p-ERK1/2, ERK1/2, p-p90RSK, Bax, Bcl-2, CDC2, CDK4, CDK6, Cyclin B1 and Cyclin D1 were measured by specific antibodies with GAPDH as the loading control.

### Animal experiments

An equivalent of 1.5 × 10^6^ LN229 cells was injected in situ into the right brain of nude mice as described in a previous study. After 7 days of implantation, tumor-carrying mice were randomly divided into three groups (n = 10). The drug treatment was as follows: Vehicle group with PBS, 40 µM Mito-LND group, and 80 µM Mito-LND group (5 µL/mouse). All treatments were performed intracranial in situ once a week, a total of four times. After 4 weeks, 3 mice in each group were randomly euthanized, followed by cerebral perfusion was performed and hematoxylin and eosin (H&E) staining to evaluate the tumor size. The remaining 7 mice in each group were used for survival analysis. The mice with neurological symptoms, such as rotational behavior, reduced movement, and dome-shaped heads caused by tumor progression, were euthanized.

### Histopathological analysis

The whole brains of mice in the three groups were fixed overnight in 4% paraformaldehyde, embedded in paraffin, serially sliced into 5-µm sections, fixed on glass slides, and dried in an oven. For H&E staining, the sections were dewaxed in xylene, hydrated with graded alcohol, and rinsed under running water. After staining for 5 min, the sections were dehydrated and sealed with neutral gum. The images were captured under a light microscope (Leica).

### Statistical analysis

Each experiment was repeated more than 3 times independently. The selected chart was one of the results of repeated experiments. The experimental results were statistically analyzed by using the statistical software GraphPad Prism 6.0. Data were represented as mean ± standard deviation. Comparison between two groups was analyzed by unpaired Student’s *t* test. One-way analysis of variance (ANOVA) was used for the comparison more than two groups. Kaplan-Meier method was used for survival analysis. Log-rank Test was used to compare whether there was a difference in survival time between the two groups. α = 0.05 was determined as the test level. *P < 0.05 was considered as statistical significance in all results.

## Results

### Mito-LND significantly inhibits the proliferation and colony formation of GBM cells

**Fig. 1:**
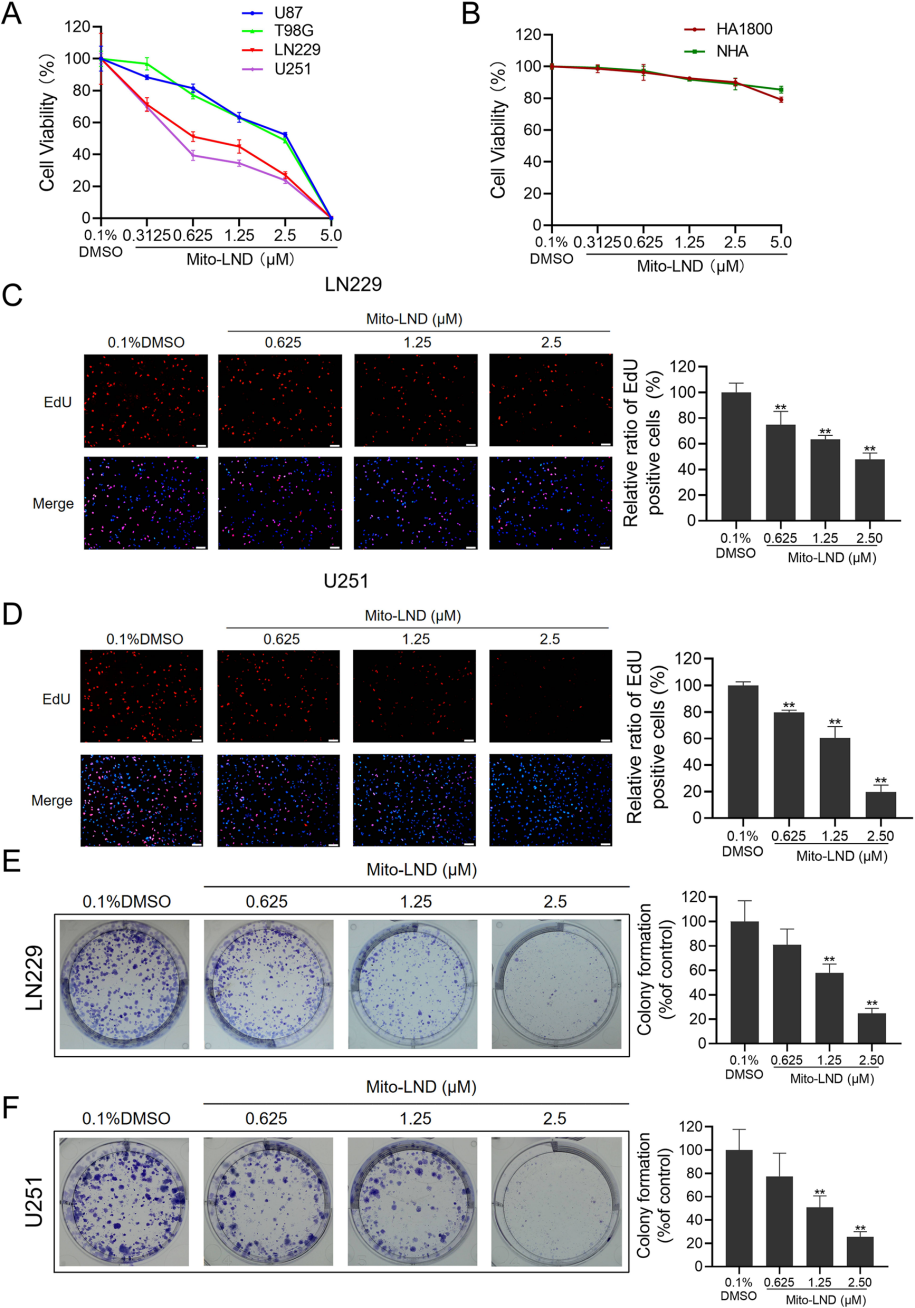
Mito-LND significantly inhibits the survival and proliferation of GBM cells. **A**, **B** GBM and normal human astrocyte cells were treated with 0.1% DMSO or indicated concentrations of Mito-LND for 72 h. The cell viability was measured using CCK-8 assay. **C**, **D** Representative images from the EdU analysis of cell proliferation after treatment of LN229 and U251 cells with Mito-LND, Scale bar: 100 μm. Quantitative results of EdU incorporation assay were analyzed. **E**, **F** LN229 and U251 cells were treated with indicated concentration of Mito-LND for 12 h, and then changed with drug-free medium for another 14 days. The numbers of colony formation were counted. The numbers of colony formation were normalized to the control group. The data are presented as mean ± SD of three replicates, **P < 0.01

In order to evaluate the effect of Mito-LND on the growth of GBM cells, four types of GBM cells were selected for CCK-8 assay. The results indicated that the cell viability of the GBM cells decreased in a dose-dependent manner after 72-h treatment with Mito-LND. The IC_50_ of LN229 and U251 cells was 2.01 µM and 1.67 µM, respectively, while that of T98G and U87 cells was 3.36 µM and 3.45 µM, respectively (Fig. 1A). However, Mito-LND had no significant effects on the survival of normal human astrocyte cell lines (HA1800/NHA) at the same concentration (Fig. 1B). The existence of glioma stem cells (GSCs) is closely related to tumor recurrence and resistance to radiotherapy and chemotherapy [26]. We also examined the effects of Mito-LND on GSCs cytotoxicity and tumorsphere formation. We found that Mito-LND could significantly suppress the survival and tumorsphere formation of GSC1 and GSC2 cells. Interestingly, GSC cells were more sensitive to Mito-LND than GBM cells (Additional file 1: Fig. S1).

In order to explore the influence of Mito-LND on the proliferation of GBM, we selected LN229 and U251 cells for the EdU incorporation assay. Compared to the control group, upon Mito-LND treatment, the proliferation rates of LN229 cells had a significant decrease, and similar results were observed in U251 cells (Fig. 1C, D). In order to analyze whether Mito-LND has a long-term inhibitory effect on the growth of GBM cells, we evaluated the effect of Mito-LND on the clonogenic ability of LN229 and U251 cells by colony formation assay. The results showed that compared to the control group, Mito-LND treatment markedly inhibited the colony formation ability in both LN229 and U251 cells (Fig. 1E, F). Taken together, these results indicate that Mito-LND significantly inhibits the proliferation and colony formation of GBM cells.

### Mito-LND induces cell cycle arrest in GBM cells

**Fig. 2:**
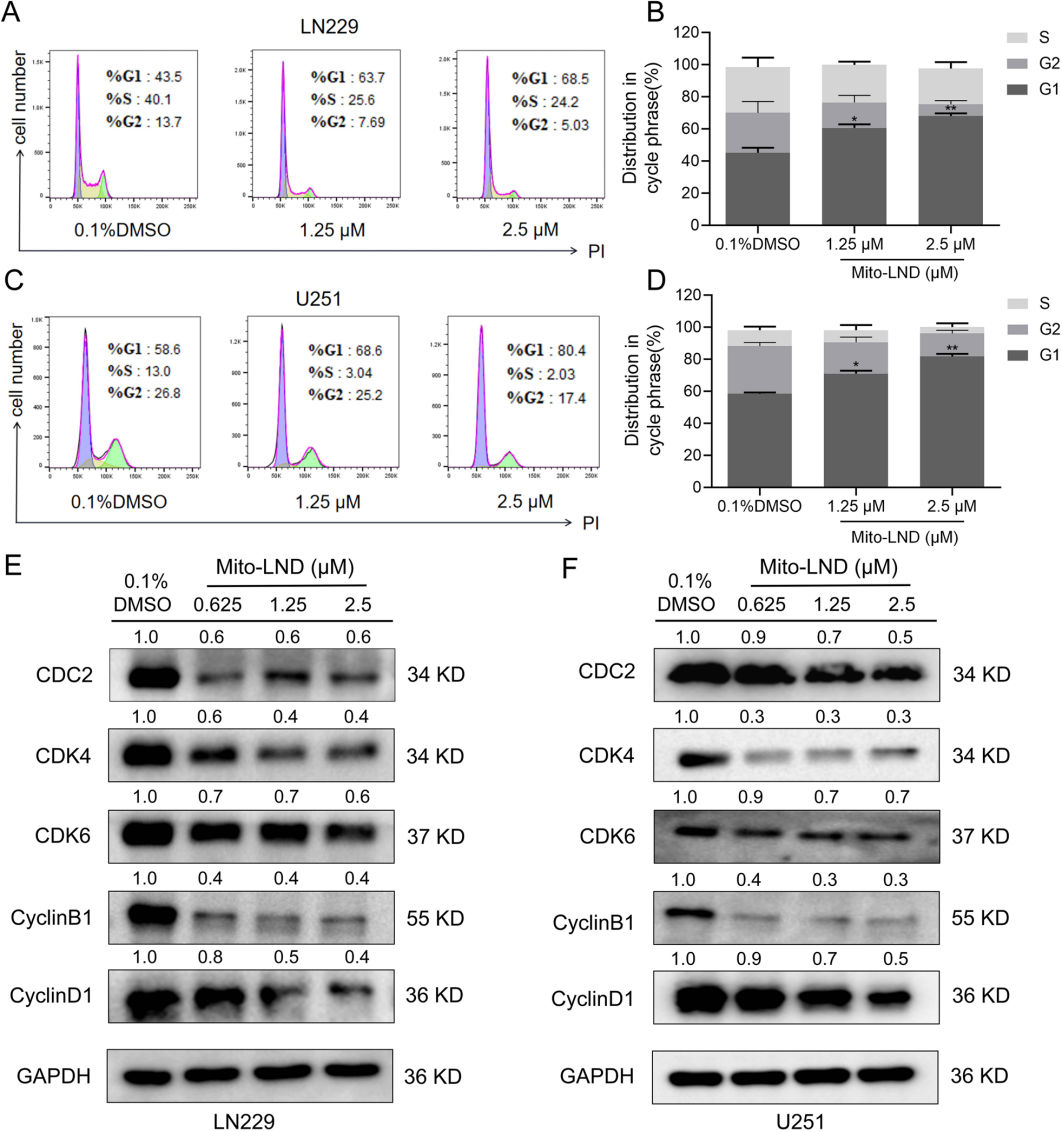
Mito-LND induces G0/G1 cell cycle arrest in GBM cells. **A** and **C** The cell cycle distribution of LN229 and U251 cells was analyzed via flow cytometry after treatment with indicated concentration of Mito-LND for 24 h, and the representative images are shown. **B** and **D** Quantitative analysis of cell cycle distribution. **E**, **F** LN229 and U251 cells were treated with Mito-LND (0-2.5 µΜ) for 24 h. The total protein was then extracted and examined through western blot analysis with the indicated antibodies. Values represent the mean ± SD, *, *P* < 0.05, **, *P* < 0.01

In order to investigate the mechanism underlying the inhibition of GBM cell proliferation by Mito-LND, flow cytometry was performed to evaluate the cell cycle distribution of LN229 and U251 cells treated with different concentrations of Mito-LND. Consequently, the proportion of LN229 cells increased significantly in the G1 phase, while the proportion in G2 and S phases decreased. Similar results were observed in U251 cells (Fig. 2A, D). These results indicated that Mito-LND could arrest GBM cells in G0/G1 phase.

Next, we examined the regulatory effect of Mito-LND on the expression of cell cycle-related proteins. Western blot results showed that the levels of CyclinB1, CDC2, CDK4, CDK6, CyclinB1 and CyclinD1 of GBM cells were downregulated after Mito-LND treatment in a dose-dependent manner (Fig. 2E, F). Taken together, Mito-LND can arrest GBM cells in the G0/G1 phase, thus inhibiting the proliferation of GBM cells.

### Mito-LND promotes apoptosis via stimulation of ROS generation

**Fig. 3:**
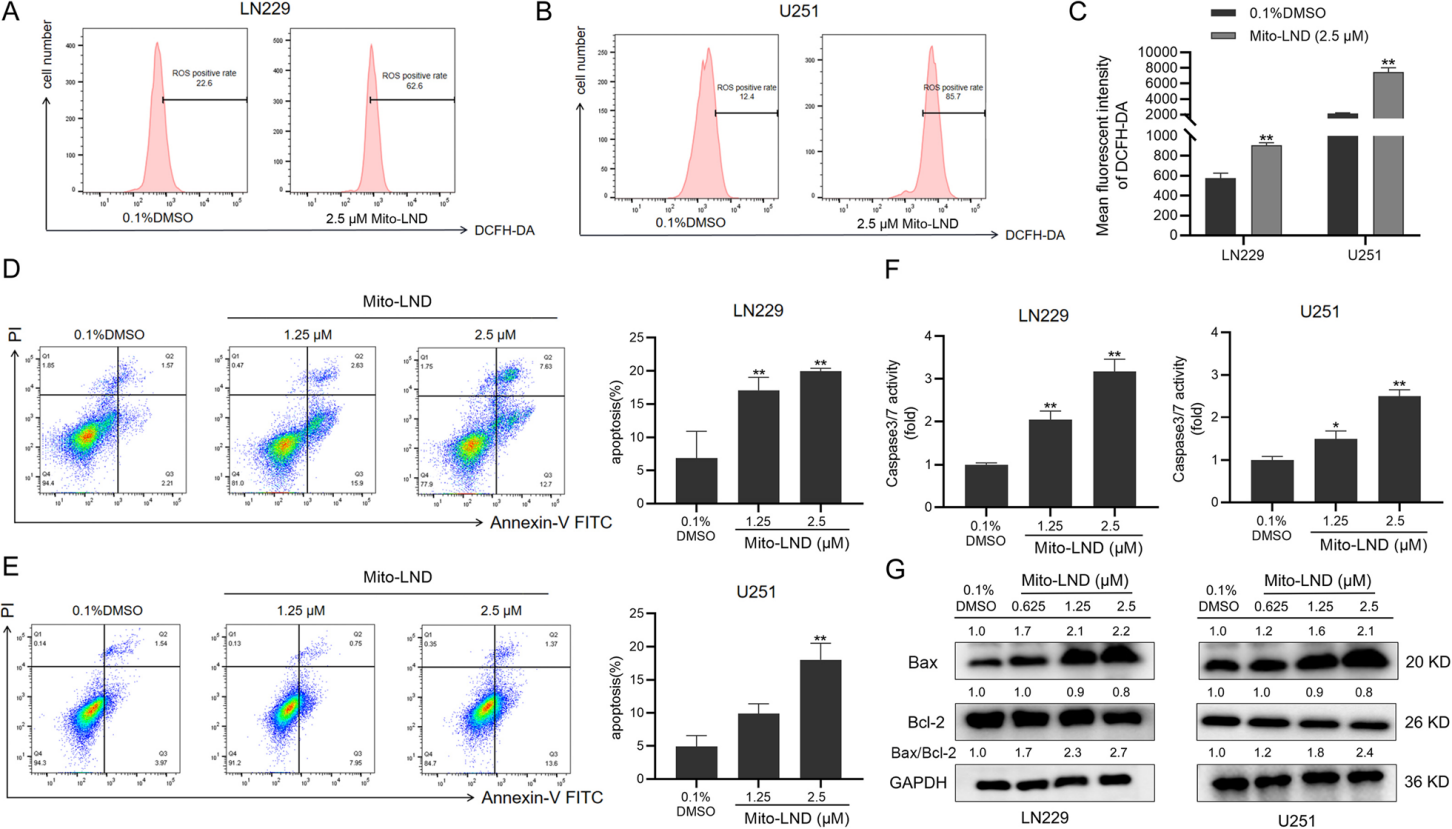
Mito-LND stimulates ROS generation and induces apoptosis in GBM cells. **A**, **B** After treatment with Mito-LND (2.5 µΜ) for 24 h, the levels of intracellular ROS in LN229 and U251 cells were measured by flow cytometry. The representative images showing ROS positive rate were showed. **C** Quantitative analysis of mean fluorescence intensity of DCFH-DA in GBM cells. **D**, **E** Cell apoptosis was analyzed by Flow cytometry in LN229 and U251 cells treated with different concentrations of Mito-LND. Quantitative analysis of the percentage of apoptosis in GBM cells. **F** Two GBM cells were treated with Mito-LND (0–2.5 µΜ) for 24 h. Caspase 3/7 activity were assessed by Caspase-Glo 3/7 activity assay. **G** The expression levels of Bax and Bcl-2 were analyzed after 24 h treatment of Mito-LND by Western blot. The data are presented as mean ± SD of three replicates (*P < 0.05, **P < 0.01)

ROS is produced naturally during various biochemical reactions in organelles [27], such as the endoplasmic reticulum, mitochondria, and peroxisome, which is also a by-product of oxygen metabolism [28]. Increased ROS levels in tumor cells can induce apoptosis and inhibit tumor cell growth [29]. Flow cytometry detected intracellular ROS levels in GBM cells and found that the intracellular ROS level was increased significantly after Mito-LND treatment (Fig. 3A, C). Furthermore, Caspase-Glo 3/7 activity kit was used to detect the activity of Caspase 3/7 in LN229 and U251 cells. The results showed that the activity of Caspase 3/7 increased gradually with the elevated concentration of Mito-LND (Fig. 3F). In order to determine whether apoptosis is related to the slow cell growth induced by Mito-LND, flow cytometry on LN229 and U251 cells revealed that the proportion of apoptotic cells increased with the elevated concentration of Mito-LND (Fig. 3D and E). To investigate the role of ROS production in Mito-LND-induced cell apoptosis, we evaluated the effect of ROS inhibitor NAC combined with Mito-LND on apoptosis. These results showed that NAC could partly rescue Mito-LND-induced cell apoptosis (Additional file 1: Fig. S2). Taken together, our data indicate that Mito-LND promotes apoptosis in GBM cells partly by inducing ROS production.

Finally, the levels of anti-apoptotic Bcl-2 and pro-apoptotic Bax in LN229 and U251 cells were assessed by Western blot. With the increase in Mito-LND concentration, the expression level of Bcl-2 was decreased, and the level of Bax was increased (Fig. 3G). Importantly, the ratio of Bax/Bcl-2 gradually increased with the increase of drug concentration, which was consistent with the increase of apoptosis by flow cytometry.

### Mito-LND inhibits mitochondrial complex I activity and reduces ATP synthesis and mitochondrial membrane potential

**Fig. 4:**
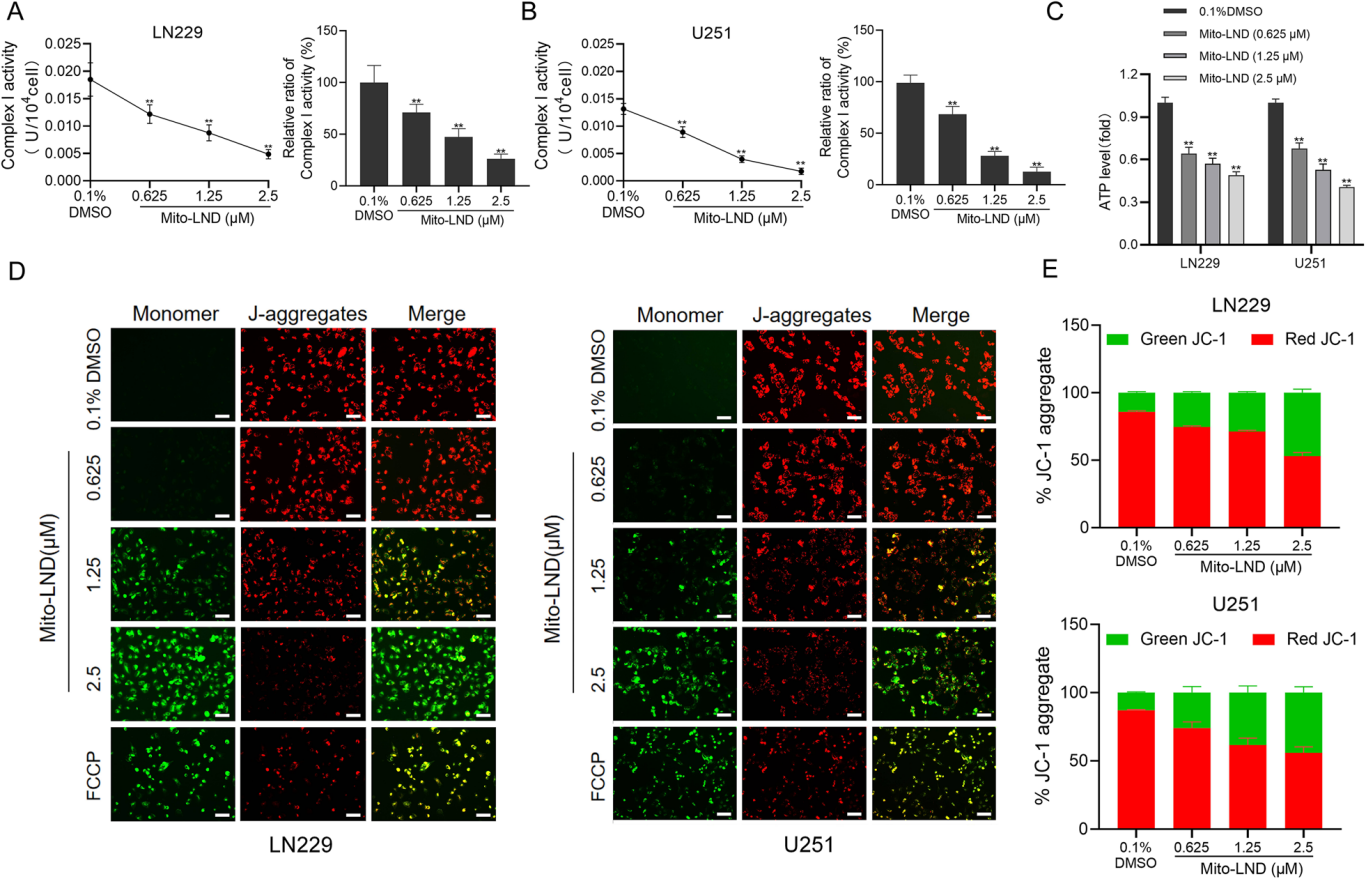
Mito-LND inhibits the activity of mitochondrial respiratory chain complex I and decreases the mitochondrial membrane potential. **A**, **B** Statistical plot of the activity of mitochondrial respiratory chain complex I in GBM cells treated with different concentrations of Mito-LND for 6 h. **C** The Changes of ATP levels in LN229 and U251 cells were measured by GloMax Luminometer. **D** Mitochondrial membrane potential changes were observed by fluorescence microscope after Mito-LND treatment. Red fluorescence indicates aggregates and green fluorescence indicates monomers, *Scale bar*: 40 μm. **E** The Changes of red and green fluorescence were measured by microplate reader. FCCP was used as a positive control in this experiment. All the data were presented as means ± SD. **P<0.01

Mito-LND acts as an inhibitor of mitochondrial complex I [24]. First, we examined the activity of mitochondrial complex I in LN229 and U251 cells after Mito-LND treatment. The activity of mitochondrial complex I in LN229 and U251 cells decreased significantly with increasing Mito-LND concentration (Fig. 4A and B). Next, we explored whether Mito-LND affects ATP synthesis and found that the intracellular ATP level of LN229 and U251 cells decreased in a dose-dependent manner after Mito-LND treatment for 24 h (Fig. 4C).

Decreased mitochondrial membrane potential is one of the first events of apoptosis, and the changes in mitochondrial membrane potential can be evaluated by JC-1 assay [30]. As shown in Fig. 4D, with the increase in Mito-LND concentration, the fluorescence intensity of red decreased while that of green increased gradually. Moreover, the changes in the ratio of red to green fluorescence in LN229 and U251 cells were quantitatively analyzed on a microplate reader (Fig. 4E), which was consistent with the results of Fig. 4D. In summary, Mito-LND inhibits the activity of mitochondrial complex I, reduces ATP synthesis, and decreases mitochondrial membrane potential.

### Mito-LND inhibits GBM cell proliferation by inactivating the Raf/MEK/ERK signaling pathway

**Fig. 5:**
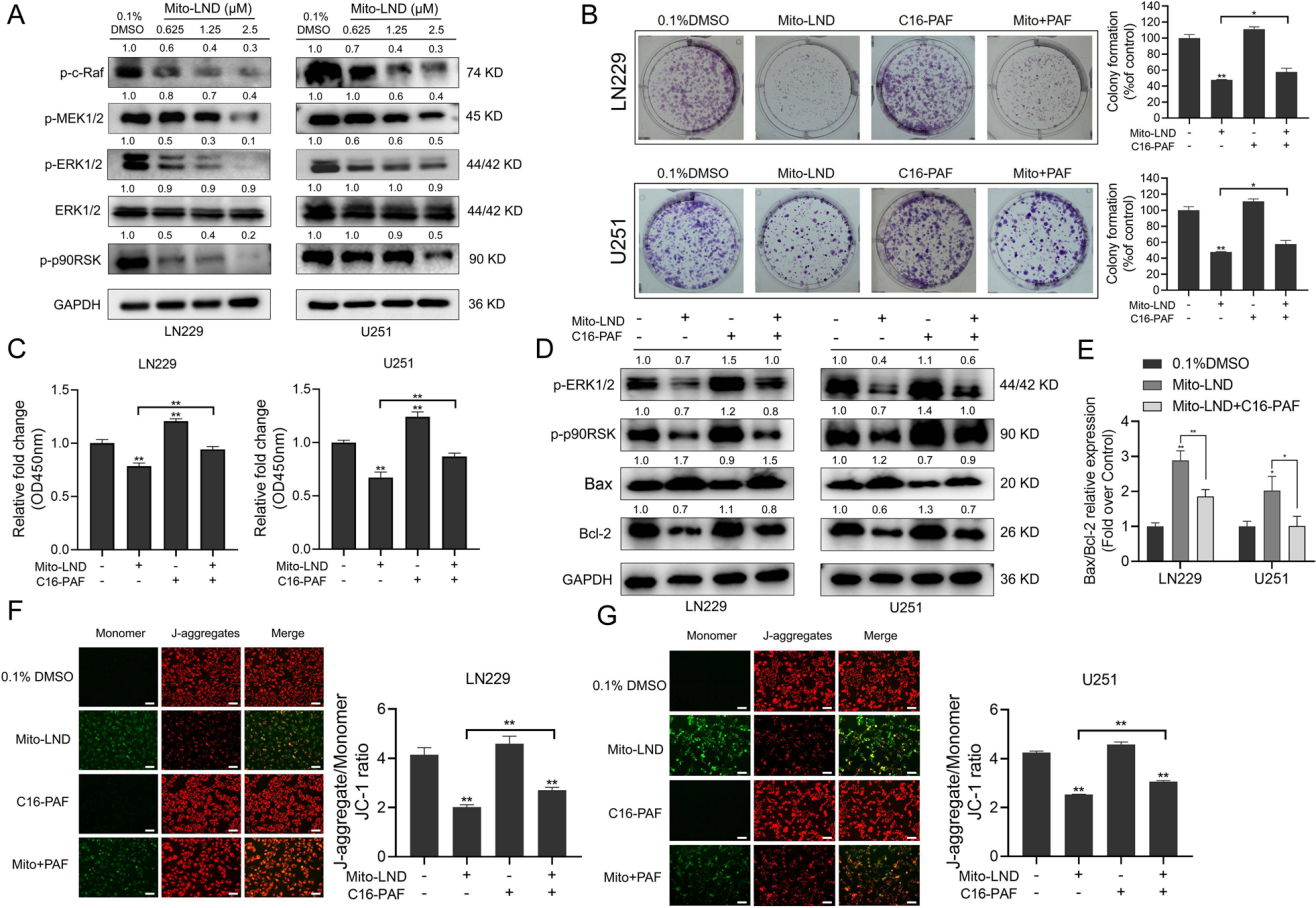
Mito-LND inhibits GBM cell growth via blocking the Raf/MEK/ERK signaling pathway. **A** The expression levels of Raf/MEK/ERK pathway-related proteins were examined after 36 h treatment of Mito-LND with indicated concentrations by Western blot assay. **B** LN229 and U251 cells were treated with 1.25 µM Mito-LND and/or 20 µM C16-PAF for 24 h. The numbers of colony formation were counted. The numbers of colony formation were normalized to that of the control group. **C** LN229 and U251 cells were treated with 1.25 µM Mito-LND and/or 20 µM C16-PAF for 36 h, then cell viabilities were evaluated by CCK-8 assay. **D** The effects of Mito-LND (1.25 µM) and/or C16-PAF (20 µM) treatment on the levels of p-ERK1/2, p-p90RSK, Bcl-2 and Bax in LN229 and U251 cells were assessed by western blot analysis. **E** The quantification results of the ratio of Bax to Bcl-2. **F**, **G** The effects of Mito-LND (1.25 µM) and/or C16-PAF (20 µM) treatment on the mitochondrial membrane potential, Scale bar: 40 μm. *P<0.05, **P<0.01

Abnormal activation of Raf/MEK/ERK signaling pathway promotes the occurrence and excessive proliferation of GBM cells [31]. It has been reported that the Raf/MEK/ERK signaling pathway blockage can inhibit the proliferation of GBM cells and induce cell apoptosis [32]. In this study, Western blot was used to detect the effect of Mito-LND treatment on the expression levels of signaling molecules in the Raf/MEK/ERK signaling pathway. The results showed that with increasing Mito-LND concentration, the expressions of p-c-Raf, p-MEK1/2, p-ERK1/2, and p-p90RSK decreased gradually, which had no effect on the protein levels of total ERK1/2 (Fig. 5A).

To further confirm the correlation between the Raf/MEK/ERK signaling pathway and inhibition of GBM cell growth by Mito-LND, we selected C16-PAF (an activator of ERK pathway) for the treatment of GBM cells. After reactivation of the ERK pathway, the inhibitory effect of Mito-LND on GBM cell proliferation was reevaluated. The colony formation assay showed that the inhibitory effect of Mito-LND on GBM cell cloning is reversed partially after C16-PAF treatment (Fig. 5B). CCK-8 results also showed that LN229 and U251 cell viability was partially rescued after C16-PAF treatment (Fig. 5C).

Furthermore, Western blot showed that C16-PAF partially reverses the inhibition of Mito-LND on the expression levels of p-ERK1/2 and its downstream related proteins (Fig. 5D). Also, the expression level of anti-apoptotic Bcl-2 was increased, and pro-apoptotic Bax level was decreased after the reactivation of the Raf/MEK/ERK pathway. Co-treatment of C16-PAF with Mito-LND inhibited the Mito-LND-induced increase in the ratio of Bax to Bcl-2 (Fig. 5E). JC-1 assay showed that mitochondrial membrane potential was restored partially after reactivation of the Raf/MEK/ERK pathway (Fig. 5F and G). Taken together, these findings indicate that Mito-LND inhibits the proliferation of GBM cells by partially blocking the Raf/MEK/ERK signaling pathway.

#### Mito-LND inhibits the growth of GBM xenograft tumors in mice

**Fig. 6:**
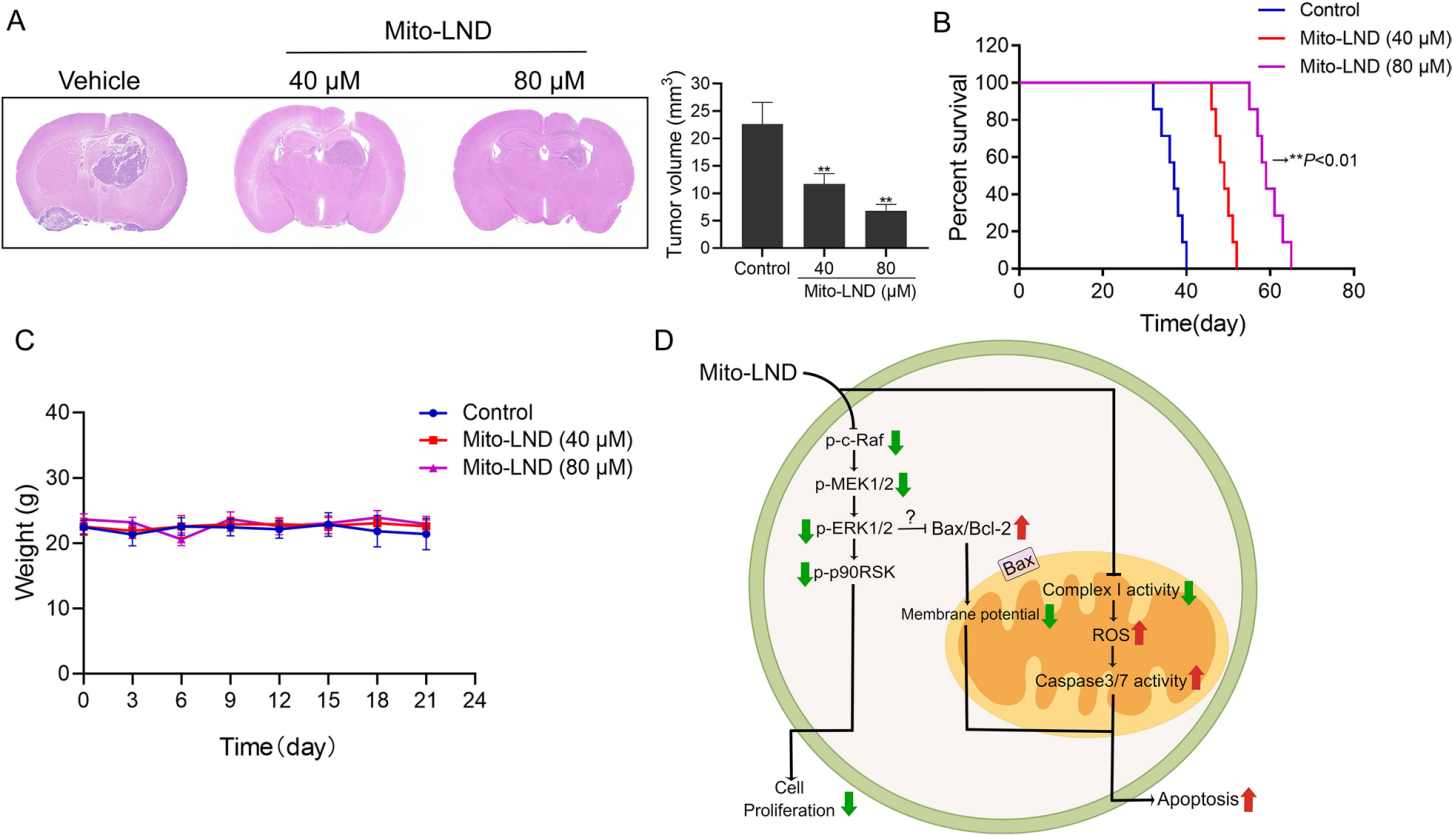
Mito-LND inhibits the GBM growth in vivo and prolonged survival time of tumor-bearing mice. **A** Representative images and statistical analysis of H&E staining of whole brain sections from control and Mito-LND treatment groups. **B** Survival analysis of mice in the control and Mito-LND treated groups (**P < 0.01). **C** Changes of mice body weight were recorded after Mito-LND administration. **D** Working model of the GBM growth inhibition by Mito-LND. Mito-LND can inhibit the glioma growth via blocking the Raf/MEK/ERK signal pathway. Altering the Bax/Bcl-2 ratio induces the decrease of mitochondrial membrane potential. Meanwhile, Mito-LND inhibits the activity of mitochondrial respiratory chain complex I and increases ROS generation, thus further inducing cell apoptosis

In order to further confirm the efficacy of Mito-LND on GBM cells, we constructed an orthotopic xenograft tumor model of LN229, which was treated with different doses of Mito-LND. H&E staining of brains showed that the intracranial tumor volume of nude mice treated with Mito-LND was significantly smaller than that in the vehicle group (Fig. 6A). Survival analysis results showed that Mito-LND treatment could significantly prolong the median survival time of tumor-bearing mice. Compared to the vehicle group, the median survival of mice treated with 40 µM and 80 µM Mito-LND was increased by 12 and 22 days, respectively (Fig. 6B). In addition, body weight of mice was not affected by Mito-LND treatment (Fig. 6C). In summary, Mito-LND inhibits the growth of intracranial GBM in mice and prolongs the median survival time of tumor-bearing mice.

## Discussion

Glioblastoma (GBM) is the most aggressive and fatal brain tumor of the central nervous system, with an extremely low 5-year survival rate [33]. It is highly invasive and protected by the blood-brain barrier (BBB), which is challenging for the treatment [34]. Interestingly, we find that Mito-LND targeting mitochondrial complex I significantly inhibits the proliferation of GBM cells and delays the growth of intracranial tumors in mice. Moreover, Mito-LND inhibits the activity of mitochondrial complex I and reduces mitochondrial membrane potential, thus stimulating the excess production of ROS and inducing tumor cell apoptosis. Importantly, Mito-LND affects GBM cell proliferation by inhibiting the activation of the Raf/MEK/ERK signaling pathway.

Mitochondria is the main organelles of cells producing ATP [35]. Excessive accumulation of ROS reduces mitochondrial membrane potential, and decreases ATP production are the main causes of mitochondrial dysfunction [36]. Moreover, ROS is a by-product of cell metabolism produced by mitochondria and plays a key role in tumorigenesis [37]. Promoting ROS production to interrupt redox homeostasis is a novel strategy for the treatment of malignant tumors [38]. LND has been used in combination with other therapeutic agents to improve efficacy and overall response to cancer treatment [19, 39]. Combined LND with DOX induces glioma cell apoptosis by reducing intracellular ATP production and inducing ROS generation [40]. LND also can increase the sensitivity of GBM cells to TMZ and radiotherapy [41]. It has been shown that LND inhibits activity of respiratory complex II through inhibiting the succinate reductase. LND also induces cellular reactive oxygen species through complex II, which reduced the viability of the DB-1 melanoma cell line [42]. Chen et al. have reported that Mito-LND mainly inhibits the activity of complex I, and it is 370-fold more potent than LND for complexes I [24]. Consistently, our data indicate that Mito-LND significantly inhibits the activity of mitochondrial complex I in GBM cells, thus reducing ATP synthesis and promoting ROS production.

Apoptosis is an autonomous and orderly death controlled by genes to maintain the stability of the intracellular environment [43], which involves the activation, expression, and regulation of a series of genes [44]. The Bcl-2 protein family (including pro-apoptotic Bax, Bad, and anti-apoptotic Bcl-2, Bcl-x) plays a key role in apoptosis [45, 46 ]. The Bcl-2 protein family is mainly located in the mitochondrial outer membrane [47], and the increase in Bax/Bcl-2 ratio decreases the mitochondrial membrane potential and activates Caspase-3/7 [48]. The decreased mitochondrial membrane potential is observed before early apoptosis and is widely considered a critical marker of early apoptosis [49]. Previous studies have shown that reducing mitochondrial membrane potential can induce tumor cell apoptosis, and thus delay tumor cell growth [50]. In this study, we found that after treatment with Mito-LND, mitochondrial membrane potential decreased, and Caspase-3/7 activity increased in GBM cells, ultimately leading to cell apoptosis. Western blot also showed that the ratio of BAX to BCL-2 was increased after Mito-LND treatment. Thus, it could be speculated that Mito-LND reduces the mitochondrial membrane potential partly by promoting the increase in Bax/Bcl-2 ratio, thus promoting apoptosis in GBM cells.

ERK, a member of the mitogen-activated protein kinase (MAPK) family, is one of the critical downstream regulators of the EGFR signaling pathway [51]. Raf/MEK/ERK signaling pathway is closely related to tumorigenesis by regulating cell proliferation, apoptosis, and other biological functions [31]. Nrf2, as a pivotal transcription factor in the regulation of antioxidative stress, plays an important role in maintaining the redox balance and promote tumorigenesis [52]. KRAS/Raf signaling pathway activates the Nrf2 antioxidant system by inducing Nrf2 expression and by constitutively activating the battery of genes controlled by Nrf2 [53]. Accumulating evidence supports that Raf/MEK/ERK pathway is abnormally activated in GBM, which promotes tumor cell proliferation and inhibits cell apoptosis [54]. ERK signaling induces Nrf2 activation and regulates cell viability partly through Nrf2 in GBM cells [55]. Downregulation of Nrf2 has been shown to improve GBM sensitivity to chemotherapy drugs such as Temozolomide [56]. Interestingly, the present study demonstrated that Mito-LND is able to inactivate the Raf/MEK/ERK signaling pathway, but whether Mito-LND has an effect on Nrf2 expression still unclear. In the future research, verifying the effect of Mito-LND on Nrf2 may offer a valuable treatment for tumors with over-expressed Nrf2.

The reactivation of the ERK pathway partially reversed the inhibitory activity of Mito-LND against GBM cells and decreased the ratio of Bax to Bcl-2. Previous study showed that inactivating ERK signaling pathway could decrease the expression of Creb. Meanwhile, Creb is required for the expression of the anti-apoptotic protein Bcl-2 [57]. The specific mechanism by which the ERK signaling pathway regulates the BAX/Bcl-2 ratio has not been fully elucidated. Whether the expression of BAX and Bcl-2 is affected by the regulation of Creb expression needs to be further verified. In addition, we found that the activation of ERK improved the Mito-LND-induced decrease in mitochondrial membrane potential. Our data indicate that Bax/Bcl-2 may located downstream of the ERK pathway and also confirmed our hypothesis that changes in the Bax/Bcl-2 ratio affect the mitochondrial membrane potential.

## Conclusions

Taken together, Mito-LND has potential anti-GBM activity. Mito-LND significantly inhibits the malignant proliferation of GBM by blocking the Raf/MEK/ERK signaling pathway. Furthermore, Mito-LND induces the apoptosis of GBM cells by inhibiting mitochondrial complex I activity, reducing mitochondrial membrane potential, and promoting ROS production (Fig. 6D). In vivo experiments showed that treatment of Mito-LND prolonged the survival of tumor-bearing mice and inhibited the growth of GBM cells. Taken together, this study supports the phenomenon that targeting mitochondrial metabolism is a potential and promising strategy for GBM therapy, which will lay a theoretical foundation for future clinical trials on Mito-LND.

### Supplementary Information

**Additional file 1. Figure. S1.** Measurement of cell survival and tumorsphere formation ability after treating with Mito-LND in GSC cells. (**A**) GSC1 and GSC2 cells were treated with 0.1% DMSO or indicated concentrations of Mito-LND for 72 h. The cell viability was measured using CCK-8 assay. (**B**–**C**) GSC cells were treated with indicated concentration of Mito-LND, after 10 days, tumorspheres in each well were counted via bright-field microscopy. The number of tumorspheres were normalized to the control group. **Figure. S2.** Measurement of cell apoptosis after treating with Mito-LND and/or NAC in LN229 cells. After treatment with Mito-LND (2.5 μΜ) and/or NAC (5 mM) for 24 h, the cell apoptosis was measured by flow cytometry in LN229 cells.

## Data Availability

All datasets supporting the conclusions contained in the present report are included in the manuscript.
