# Supplementary material for: Preclinical evaluation of Mito-LND, a targeting mitochondrial metabolism inhibitor, for glioblastoma treatment
Source: J Transl Med. 2023 Aug 7;21:532. doi: 10.1186/s12967-023-04332-y (PMC10405494; doi:10.1186/s12967-023-04332-y)
Supplement: Supplementary file 1 — Additional file 1. Figure. S1. Measurement of cell survival and tumorsphere formation ability after treating with Mito-LND in GSC cells. (A) GSC1 and GSC2 cells were treated with 0.1% DMSO or indicated concentrations of Mito-LND for 72 h. The cell viability was measured using CCK-8 assay. (B–C) GSC cells were treated with indicated concentration of Mito-LND, after 10 days, tumorspheres in each well were counted via bright-field microscopy. The number of tumorspheres were normalized to the control group. Figure. S2. Measurement of cell apoptosis after treating with Mito-LND and/or NAC in LN229 cells. After treatment with Mito-LND (2.5 μΜ) and/or NAC (5 mM) for 24 h, the cell apoptosis was measured by flow cytometry in LN229 cells. [file 12967_2023_4332_MOESM1_ESM.doc]

**Supplemental data**





**Figure.S1** Measurement of cell survival and tumorsphere formation ability after treating with Mito-LND in GSC cells. (A) GSC1 and GSC2 cells were treated with 0.1% DMSO or indicated concentrations of Mito-LND for 72 h. The cell viability was measured using CCK-8 assay. (B-C) GSC cells were treated with indicated concentration of Mito-LND, after 10 days, tumorspheres in each well were counted via bright-field microscopy. The number of tumorspheres were normalized to the control group.


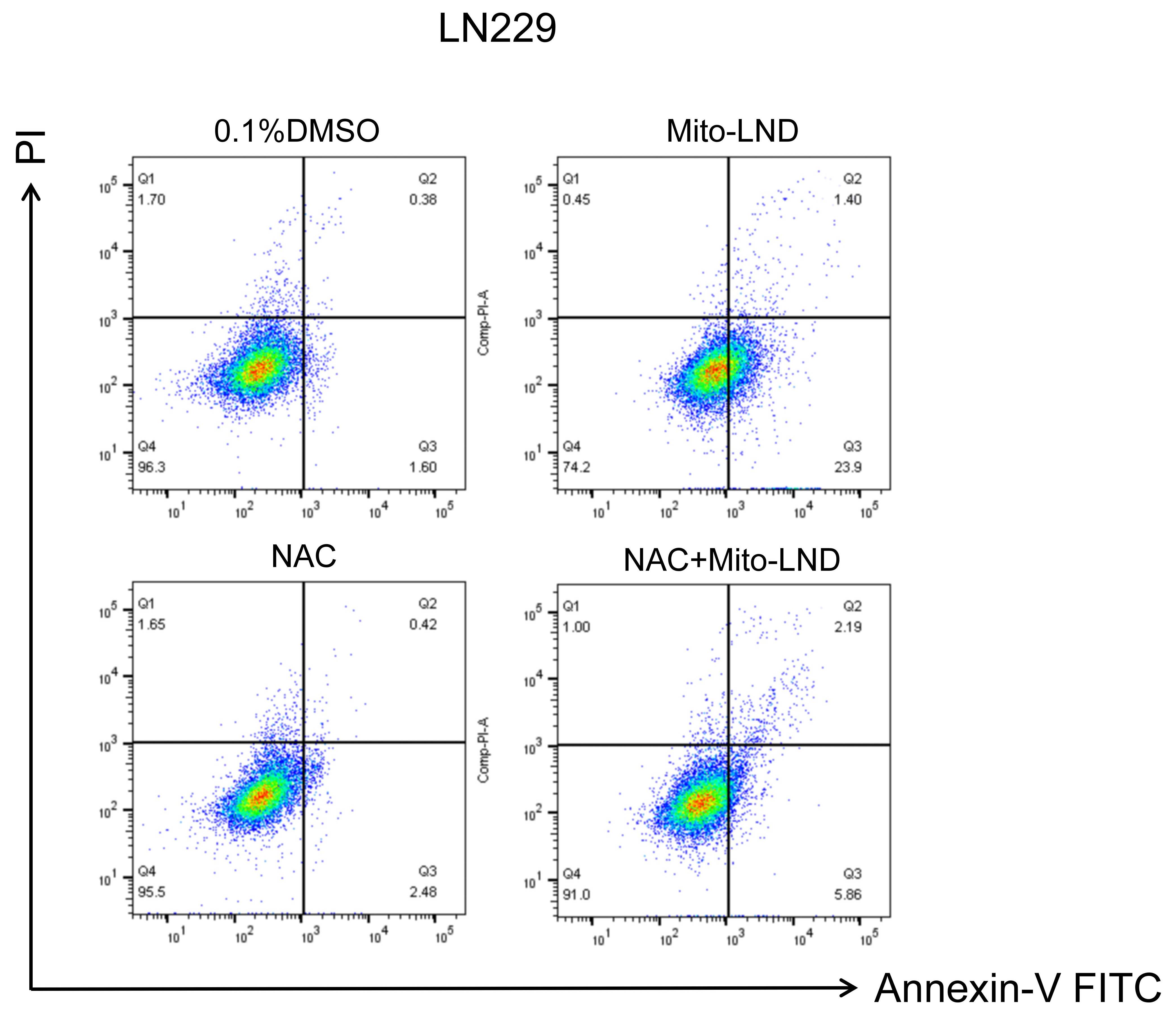


**Figure.S2** Measurement of cell apoptosis after treating with Mito-LND and/or NAC in LN229 cells. After treatment with Mito-LND (2.5 μΜ) and/or NAC (5 mM) for 24 h, the cell apoptosis was measured by flow cytometry in LN229 cells.
